# Hamstring injury risk in male professional football: do external training loads play a role?

**DOI:** 10.1136/bmjsem-2025-002649

**Published:** 2025-09-09

**Authors:** Tania Nilsson, Dan Fransson, Mats Borjesson, Matilda Lundblad, Andreas Ivarsson

**Affiliations:** 1 Department of Food and Nutrition and Sports Science, University of Gothenburg, Gothenburg, Sweden; 2 Department of Molecular and Clinical Medicine, University of Gothenburg Sahlgrenska Academy, Goteborg, Sweden; 3 Department of Medicine, Geriatrics and Acute Medicine Östra Sjukhuset, Gothenburg, Sweden; 4 Department of Orthopaedics, Sahlgrenska Academy, Goteborg, Sweden; 5 Centre of Research on Welfare, Health and Sport, Halmstad University, Halmstad, Sweden

**Keywords:** Football, Soccer, Hamstring, Prevention

## Abstract

**Objectives:**

Hamstring strain injury (HSI) is the most common time-loss injury in football and is prone to recurrence. This exploratory study aimed to describe the relationship between short-term external training load (TL) and HSI occurrence in male senior football players at the professional level.

**Methods:**

TL data in terms of global positioning system (GPS) variables and HSI incidence were collected over four seasons from 25 senior professional football players. GPS variables included total distance (TD), very intense accelerations (>3.00 m/s²), very intense decelerations (<–3.00 m/s²), high-speed running distance (>19.8 km/hour), maximal sprint distance (>29.8 km/hour) and maximal velocity. For each injury case, TL during the 7 and 14 days preceding the injury was compared with matched control periods from the same player.

**Results:**

25 hamstring injuries were included for analysis. Large interindividual variation in TL patterns was observed, with some players exhibiting higher and others lower TL in the periods preceding injury compared with control periods. No consistent group-level trends were identified between injured and control periods.

**Conclusion:**

This exploratory study found considerable individual variability in TL prior to HSI and no clear group-level patterns. These findings suggest that short-term TL metrics alone may have limited utility in predicting HSI risk at the group level in professional football players.

**WHAT IS ALREADY KNOWN ON THIS TOPIC**

- Hamstring strain injury (HSI) is the most common injury in football.
- Exposure to high-velocity training load (TL) variables has been proposed as a modifiable factor that may influence injury susceptibility.

**WHAT THIS STUDY ADDS**

- Most previous research on this topic has focused on group averages and linear statistical models. In this study, we aimed to complement such analysis with the analyse of each individual using a non-linear model.
- This study found a large individual variability and no clear trends in TL during short time periods preceding HSI in professional football players.

**HOW THIS STUDY MIGHT AFFECT RESEARCH, PRACTICE OR POLICY**

- On group level, no statistically significant difference between the periods preceding injury and the control periods was found.
- The large individual variability during the periods preceding HSI suggests that short-term TL monitoring alone might not be a reliable predictor of HSI risk.
- This study’s results highlight the complexity of injury risk assessment and support the thesis that predicting HSI risk based solely on external parameters is not feasible.
- Future research should explore more contextual factors contributing to HSI in professional football players.

## Introduction

 Hamstring strain injury (HSI) is the most common time-loss injury in football and is prone to reoccurrence.^1^ In elite male senior football, the proportion of HSI has increased from 12% to 24% during the recent decades^2^ and imposes a notable performance and economic burden.^3^ On average, each club experiences approximately 90 days of cumulative player absence and reduced availability for around 15 matches per season due to HSI.^4^

The aetiology of HSI is complex and multifactorial.^5^ Older age and a history of HSI have been identified as non-modifiable risk factors,^6^ while exposure to high-velocity training load (TL) variables such as maximal or near-maximal sprinting has been considered modifiable risk factors.^57^,^9^ The majority of HSIs occur during rapid movements such as sprint acceleration and high-to-maximal velocity running, where high eccentric demands are placed on the posterior thigh.^7 10 11^ Increased match demands and exposure to high-velocity running might elevate the risk of sustaining an HSI.^2 12^ Examining the association between HSI and high-velocity TL variables is therefore of interest to practitioners striving to reduce HSI risk for football players.

Previous research has shown that a 30-day period with low and/or increasing average TL volume might be a period when players have an increased risk of overall injury in professional youth football.^13^ Similar results have been found in previous studies in professional football.^14 15^ However, many of these studies rely on group-level data, use linear statistical models and evaluate TL over relatively long periods, which may fail to capture individual variability and acute TL changes relevant to decision-making in real-world settings.^16 17^ This might limit their applicability in environments where coaches and medical teams need to assess risk and adjust training on a weekly or even daily basis.

More scientific information is warranted investigating shorter time periods regarding TL variability and its relation to HSI risk in professional football. This study therefore focuses on shorter time frames, specifically 7 and 14 days, to reflect the real-world cadence of training interventions, especially in high-performance settings where microcycles (eg, Monday-to-Sunday blocks) are standard. Recent studies have suggested that non-linear models might be beneficial to discover relationships between TL and injury.^18^ In contrast to broader workload metrics such as the acute:chronic workload ratio, which compare rolling averages over weeks, this approach seeks to identify immediate fluctuations that could inform short-term adjustments. Because the majority of previous injury research on this topic has focused on group averages and linear statistical models, in this study, we aimed to combine group-level analyses with individual non-linear models. The overall aim of this study was to explore average levels, absolute values and variability in external TL during short periods prior to an HSI in a group of Swedish male professional football players.

## Methods

The study was designed as an observational cohort study. Data were collected over four seasons. A 7-day period is commonly used as a periodisation model in football to facilitate TL planning leading up to match, while a 14-day period, often referred to as a mesocycle, is used to provide a slightly longer timeframe for planning.^19^ Therefore, all TL variables were analysed by dividing individual players’ TL data into 7-day and 14-day periods preceding an HSI, including the day of injury, collectively referred to as ‘periods preceding injury’. The two periods preceding injury were compared with two equally long control periods. The control period for each individual player was selected as the 7-day or 14-day period directly before the 7-day or 14-day period preceding HSI. This structural model was chosen with the aim to have an individual aspect through all of the statistical analysis, and to be able to reach the goal of analysing shorter time periods of TL and its relation to HSI risk in professional football.

### Participants

In total, 25 senior players (age: 26±4 years, stature: 181.0±6.8 cm, body mass: 74.0±7.4 kg) from three different professional clubs in the Swedish 1st division were included in the study. The participant distribution across clubs was 60%, 32% and 8%, respectively. Baseline data were collected at the time of player inclusion. All outfield playing positions were represented (seven defenders, nine midfielders, nine attackers). Exclusion criteria included goalkeepers (due to their distinct activity profiles), players with missing or unreliable TL data prior to injury and cases classified as reinjuries within the same season. The number of participants was considered sufficient for the analyses conducted.

### Injury definition

The study design concerning injuries followed the consensus on definitions and data collection procedures in studies of football injuries according to Waldén *et al*.^20^ The teams’ medical staff prospectively recorded HSI after each training and match, using a standard injury form. A player was considered injured when he had any absence from future football participation (time-loss injury).^21^

### TL quantification

Global positioning system (GPS) derived TL was quantified during all outfield training sessions and matches using 10 Hz GPS (Catapult Vector S7; Catapult Innovations, Melbourne, Australia), which has demonstrated strong validity and reliability for quantifying movement metrics in team sports.^22 23^ Interunit reliability has also been reported as high (coefficient of variation 1.7%–6.3%).^23^ The units were placed between the players’ shoulders and worn by the same player in all sessions and matches. Data sets were verified for the number of satellites connected (mean>11) and horizontal dilution of precision (mean<1.2) before being included in the analysis.^24^

HSI most commonly occurs during rapid, high-intensity actions.^7^ Accordingly, GPS variables related to intense running were selected for this study (table 1) based on their relevance to football TL and potential association with injury risk.^7 25 26^ In cases of missing data (eg, GPS malfunction or non-use), individual TL averages were used, in line with previous research.^27 28^

**Table 1:** Description of GPS variables and speed zones

Variable	Unit	Zone
Total distance (TD)	m	TD covered including all movement (>0 km/hour)
Very intense accelerations	N	Number of times accelerating above 3.00 m/s^2^
Very intense decelerations	N	Number of times decelerating below −3.00 m/s^2^
High-speed running distance	m	Distance covered above 19.8 km/hour
Maximal sprint distance	m	Distance covered above 29.8 km/hour
Maximal velocity	km/hour	Highest speed reached during activity

GPS, global positioning systems.

### Statistical analysis

Descriptive analyses were performed using JASP V.0.19.0. For all analyses, we analysed potential differences in TL between the baseline (15–28 days before the injury, ‘control period’) and the ‘period preceding injury’ (1–14 days before the injury occurred). To compare the average level of the TL measures between the different time periods, linear mixture models, using JASP (V.0.19.0, JASP Team, 2025), were used. In these analyses, players were included as a random effect variable to account for the nested data structure. The two different periods were included as a fixed effect parameter. For these analyses, we considered a p<0.05 to indicate a statistically significant result.

To analyse the absolute values and individual variability between the periods preceding injury and the control periods, we calculated non-overlap of all pairs (NAP). NAP is a non-parametric technique for measuring non-overlap or ‘dominance’ for two phases and a feasible way to interpret individual effects between two periods. An NAP equal to 0.5 indicates no effect (chance overlap), while any value >0.5 is equal to a positive effect and any value <0.5 is an undesirable effect.^29^ This study, based on the recommendations by Parker and Vannest,^29^ used the following benchmarks to interpret the results; 0–0.07 (strong effect in favour of sequence A), 0.08–0.34 (moderate effect in favour of sequence A), 0.35–0.65 (no systematic effect for any of the sequences), 0.66–0.92 (moderate effect in favour of sequence B) and 0.93–1.00 (strong effect in favour of sequence B).

The advantages of NAP are that it can be applied in distributions that lack normality and that all data points collected are included in the analyses. The disadvantages are that it cannot be used to evaluate trends or serial dependency. For a more thorough explanation of NAP and its application, see Parker and Vannest.^29^ The NAP analyses were conducted in R using the SingleCaseES package (V.0.7.3).^30^ Results are presented both on the overall and individual levels.

### Patient and public involvement

Players participated in their own team’s activities each week and nothing more. The participating clubs received a written report on the study results.

### Equality, diversity and inclusion statement

The study involved male professional senior football players from three clubs in southern Sweden. The sample did not include amateur-level players or individuals from other regions. The research team comprised five members (two women and three men) with expertise in physiology, medicine and psychology.

## Results

A total of 35 hamstring injuries were recorded during the study period, of which 10 were excluded due to missing TL data. Consequently, 25 injuries were included in the final analysis. Five injuries (20%) occurred in players with a history of previous hamstring injury. The mean age of the injured players was 26±4 years.

**Figure 1:**
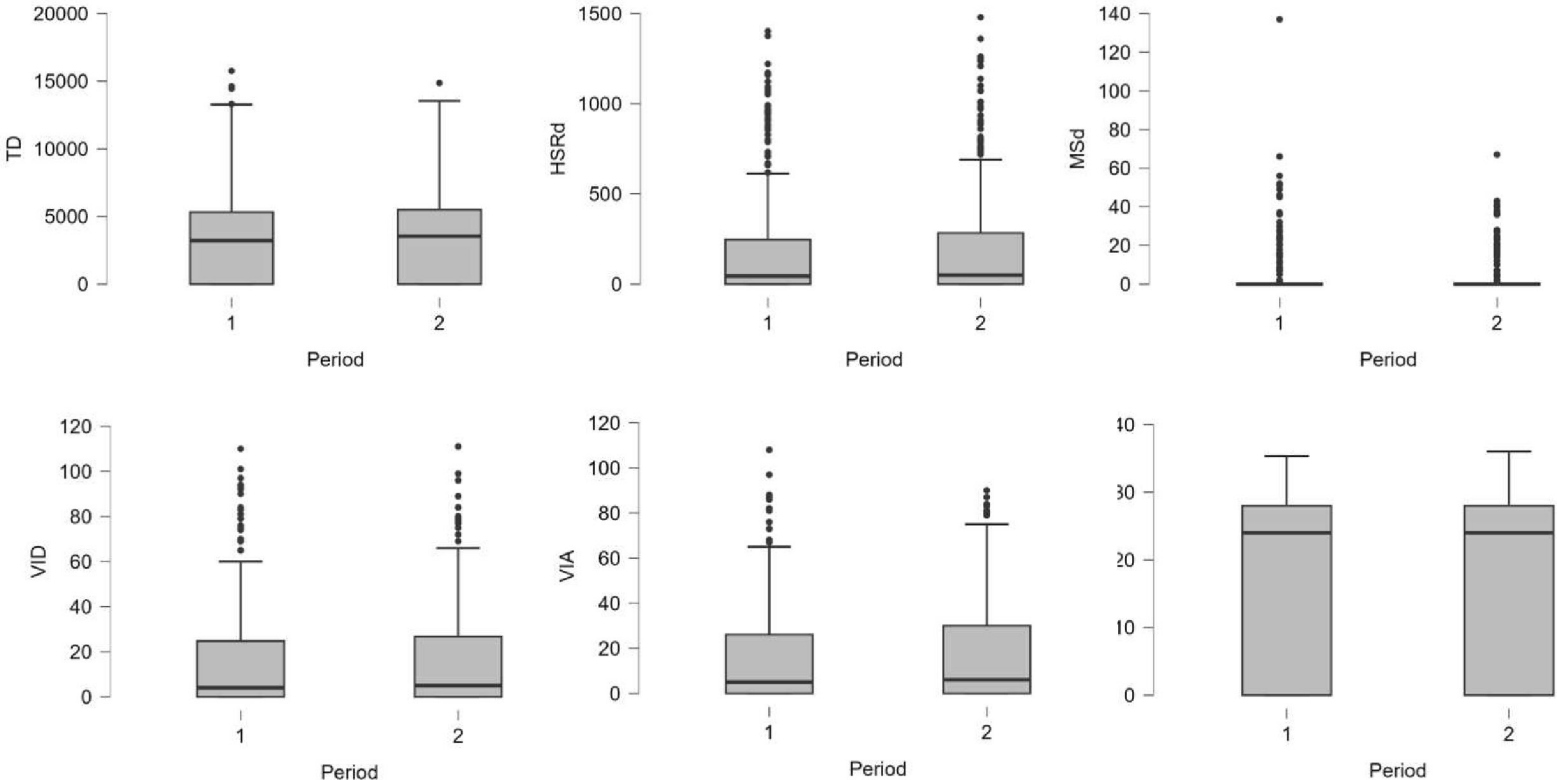
Boxplots illustrating individual-level data for total distance (TD), high-speed running distance (HSRd), maximal sprint distance (MSd), very intense decelerations (VID), very intense accelerations (VIA) and maximal velocity (VMax) during the 14-day periods preceding hamstring strain injury (period 1) and their matched control periods (period 2).

The linear mixed model analyses revealed no statistically significant differences in any of the TL parameters between the periods preceding injury and their corresponding control periods. Descriptive statistics are provided in table 2 and individual-level data in boxplots (figures1^2^). At the group level, NAP values indicated no consistent differences in TL during the 7-day or 14-day periods prior to injury (NAP range: 0.45–0.54). However, considerable interindividual variability was observed, with TL values both exceeding and falling below those of the control periods in the short-term windows preceding injury (tables3  4). Complete individual NAP values are available in the online supplemental material.

**Table 2:** Training load data in periods preceding hamstring strain injury and control periods

		Control period		Period preceding injury		P value
		Median (per day)	IQR	Median (per day)	IQR	
TD	7 days	3354.50	5553.75	3055.50	4984.00	0.33
	14 days	3537.00	5499.00	3205.50	5318.25	0.84
HSRd	7 days	61.50	288.50	35.50	210.25	0.31
	14 days	49.50	284.00	44.50	246.00	0.61
MSd	7 days	0.00	0.00	0.00	0.00	0.90
	14 days	0.00	0.00	0.00	0.00	0.41
VID	7 days	4.50	25.75	4.00	23.25	0.44
	14 days	5.00	26.75	4.00	24.75	0.72
VIA	7 days	6.50	27.50	4.50	26.00	0.72
	14 days	6.00	30.00	5.00	26.00	0.46
VMax	7 days	25.00	28.00	24.00	27.00	0.69
	14 days	24.00	28.00	24.00	28.00	0.94

Training load data across all variables for the 7-day and 14-day periods preceding hamstrings strain injury and control periods. Data presented as median and IQR; significant differences between periods preceding injury and control periods (p<0.05).

HSRd, high-speed running distance; MSd, maximal sprint distance; TD, total distance; VIA, very intense accelerations; VID, very intense decelerations; VMax, maximal velocity.

**Table 3:** NAP values and interpretation—7-day periods preceding hamstring strain injury

Variable	NAP	Interpretation	>0.5	<0.5
TD	0.48	No difference	10	15
HSRd	0.47	No difference	11	14
MSd	0.46	No difference	11	14
VID	0.49	No difference	13	12
VIA	0.48	No difference	12	13
VMax	0.45	No difference	8	17

NAP values across all variables for the 7-day period before injury.

HSRd, high-speed running distance; MSd, maximal sprint distance; NAP, non-overlap of all pairs; TD, total distance; VIA, very intense accelerations; VID, very intense decelerations; VMax, maximal velocity.

**Table 4:** NAP values and interpretation—14-day periods preceding hamstring strain injury

Variable	NAP	Interpretation	>0.5	<0.5
TD	0.48	No difference	10	15
HSRd	0.51	No difference	14	11
MSd	0.54	No difference	17	8
VID	0.49	No difference	12	13
VIA	0.50	No difference	11	14
VMax	0.51	No difference	13	12

NAP values across all variables for the 14-day period before injury.

HSRd, high-speed running distance; MSd, maximal sprint distance; NAP, non-overlap of all pairs; TD, total distance; VIA, very intense accelerations; VID, very intense decelerations; VMax, maximal velocity.

**Figure 2:**
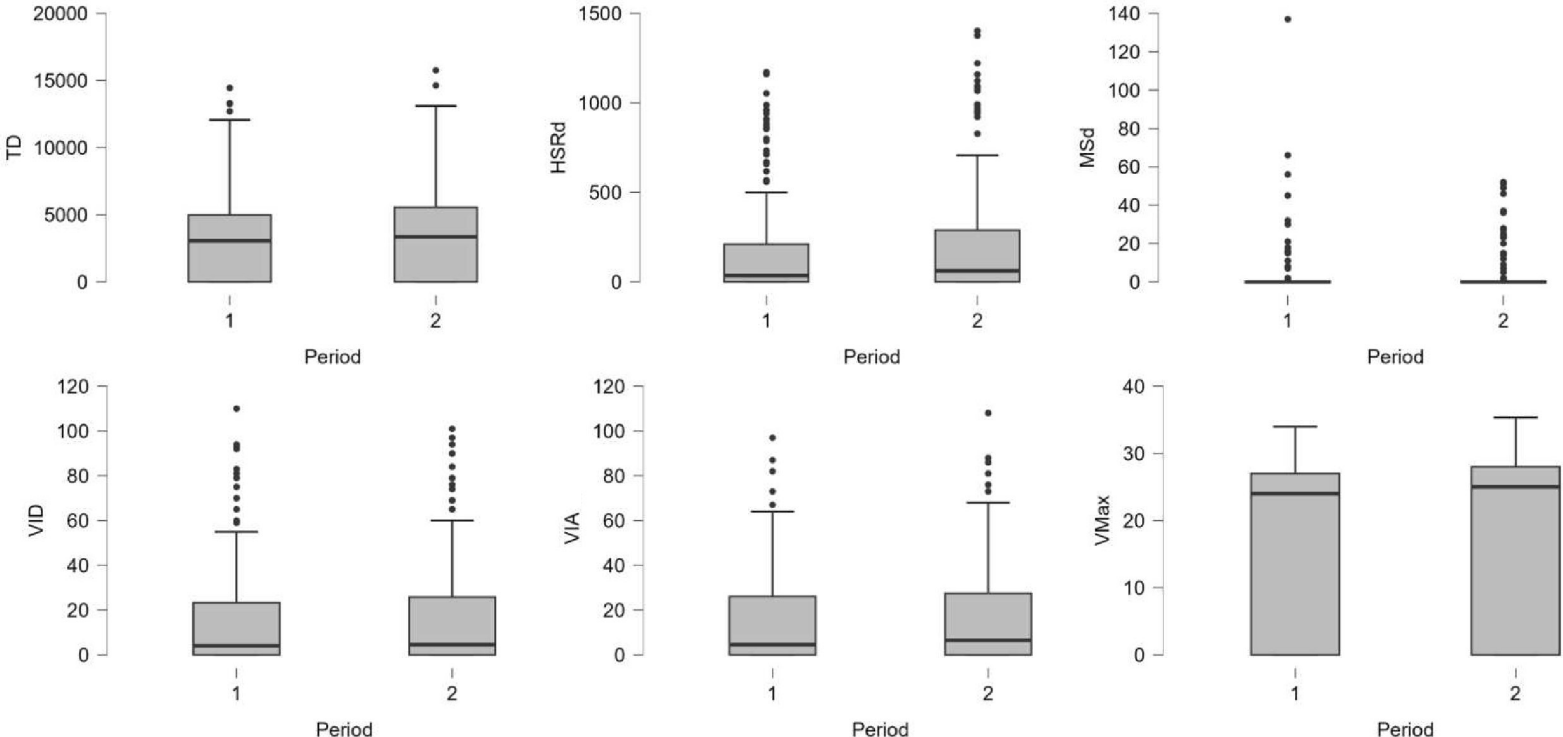
Boxplots illustrating individual-level data for total distance (TD), high-speed running distance (HSRd), maximal sprint distance (MSd), very intense decelerations (VID), very intense accelerations (VIA) and maximal velocity (VMax) during the 7-day periods preceding hamstring strain injury (period 1) and their matched control periods (period 2).

## Discussion

TL from a 7-day and a 14-day period preceding HSI was compared with TL from control periods of 7 and 14 days, respectively. This analysis aimed to compare whether there were any systematic differences in load on the days before injury in comparison to a control period. The key finding of this study was that there was a considerable variability in TL among players, in the periods preceding HSI, with TL values fluctuating both above and below those of the control periods. However, at the group level, no systematic differences were observed between the time periods.

Previous research has indicated that a 30-day period characterised by low and/or increasing average TL may be associated with an elevated risk of overall injury.^13^ Complementing these findings, a more recent study reported that players with an HSI exhibited greater external load fluctuations during a 28-day period preceding injury compared with a preinjury reference block, across all examined load variables.^15^ The present study focused on shorter time periods of TL variability and its relation to HSI risk in professional male football. Shorter time periods may be more relevant in practical settings than longer time periods, possibly helping to make informed decisions regarding TL adjustments more frequent and may also yield more specific results compared with when analysing longer periods. However, the current study did not reveal any clear trends or statistically significant differences when examining group averages for any of the TL variables in the 7-day and 14-day periods preceding HSI, compared with control periods.

Interindividually, there was a considerable variation in all TL variables during the periods preceding injury, with TL values distributed both higher and lower compared with the control periods. The range of NAP values in the 7-day period varied as follows: total distance (TD: 0.29–0.65), high-speed running distance (HSRd: 0.27–0.65), very intense decelerations (VID: 0.24–0.67), very intense accelerations (VIA: 0.27–0.67) and maximal velocity (VMax: 0.18–0.63). Similarly, in the 14-day period, TD ranged between 0.29 and 0.65, HSRd between 0.31 and 0.64, VID between 0.31 and 0.64, VIA between 0.31 and 0.70 and VMax between 0.33 and 0.66. The variation was smallest for the variable maximal sprint distance (MSd) in both the 7-day and 14-day periods, with NAP values ranging from 0.36 to 0.57 and 0.43 to 0.57, respectively. There was a relatively small amount of MSd produced for each session, in both the periods preceding an HSI and the control periods. The scarcity of MSd data is likely due to the high-speed threshold set for this variable (>29.8 km/hour), which results in players accumulating only a minimal amount of data each week, potentially not even in every training session. The low variation in MSd across both time periods indicates that players tended to accumulate a similar amount of MSd in both the periods preceding HSI and the control periods. In summary, our findings demonstrate that there was no systematic pattern in whether external TL is higher or lower during a short time period before the injury event, compared with control periods. These findings do not negate the potential importance of TL but rather suggest that TL alone may not fully explain injury occurrence, particularly over short time frames, and highlight the complexity of HSI risk.

It is commonly argued that an excessive overload or a rapid increase in TL can lead to inadequate adaptations, where the strength of the tissues may be surpassed, thereby increasing the risk of injury or causing tissue damage.^31^,^33^ If a player can train hard in a safe way over a longer period of time, the player may develop a better tolerance to specific loading and performance requirements, potentially resulting in tissues that are more robust and less susceptible to injuries.^32^ The primary objective of TL monitoring might thus be to ensure that athletes are following the training plan and to assess how well athletes are adapting to the TL over time. Evaluating TL from an individual perspective might therefore serve as one tool to evaluate the players’ robustness against injury as part of a multifactorial assessment and might be seen as an indicator of a generally increased risk of injury rather than a predictor of injury.

While our study did not reveal any clear TL patterns during the investigated short time periods preceding HSI, previous research has suggested that even shorter periods of high-velocity running, such as sprinting more than 30 m within 5 min, may increase the likelihood of HSI during matches.^34^ The total TL accumulated over an entire training session may not capture potential injury-related TL patterns occurring within shorter time frames. Future research focusing on running velocities during brief time windows (eg, 1–15 min) immediately before an HSI might provide a more detailed description of acute accumulated TL in relation to HSI risk. Such analyses could help identify specific TL patterns preceding HSI that may be relevant for prevention strategies.

Previously published research highlights the complex nature of injury prevention and emphasise the importance of adopting more comprehensive methods and multimodal interventions when trying to improve injury prevention.^35 36^ Impellizzeri *et al* have previously suggested that an excessive focus on TL might divert attention from the complex, multifactorial causes of injury and overlook the significance of other key contextual factors.^37^ Our findings support this perspective by reinforcing the idea that TL should not be interpreted in isolation, but rather in combination with other relevant factors. Multiple factors that might contribute to injury should be taken into consideration and used to make the best-informed decisions regarding the individual player. For example, accumulated fatigue has been suggested as a factor of relevance in HSI injury prevention, since neuromuscular accumulated fatigue might subsequently increase the risk of overall injury.^38^ Also, sprint mechanics have been suggested as a potential contributing factor to HSI.^39^ To get a broader picture of HSI risk in professional football players, future research could focus on sprint mechanics, fatigue and recovery status measures. This type of information may then be used to evaluate the relationship with potential HSI and considered alongside TL metrics in multifactorial risk profiling.

### Clinical implications

Although no systematic trends were observed at the group level in this study, the pronounced interindividual variability in TL before injury highlights the importance of individualised monitoring in professional football settings. In practical terms, individualised monitoring involves establishing a baseline TL profile for each player, accounting for their historical exposure, positional demands and physical characteristics. This can be achieved through consistent tracking of key TL metrics and identifying each player’s typical weekly fluctuations and thresholds. Rather than comparing players to group averages, practitioners could assess deviations from each player’s normal TL patterns, especially sharp increases or decreases in TL, as potential red flags. Furthermore, incorporating subjective data (eg, perceived exertion, wellness scores, fatigue measurements) and contextual information (eg, recent match exposure, travel, recovery time) allows for a more comprehensive and responsive load management strategy. The results of this study underscore that short-term fluctuations in TL may be insufficient to predict injury on their own, but when interpreted in the context of individualised norms, they may still contribute meaningfully to informed decision-making in injury prevention strategies.

### Limitations

This type of research presents methodological challenges, and several limitations should be acknowledged.^18 40^ The mechanisms behind HSI are likely multifactorial, involving factors such as age, injury history, strength, biomechanical function, fatigue and phase of the running cycle. Although relevant, these factors could not be fully controlled in this study. Only time-loss injuries were included, which may have understated the load–symptom relationship by omitting subclinical or non-time-loss complaints. Another limitation of this study is the small sample size, with only 25 injuries included. This limits the extent to which the findings can be generalised to other populations. Moreover, the findings are limited to male professional footballers and may not generalise to other populations. Future studies with larger cohorts are needed to validate these results and assess their applicability across different settings.

## Conclusion

This study investigated external TL during 7-day and 14-day periods preceding HSIs in professional football. No systematic differences in TL were found at the group level and considerable interindividual variation was observed during the 7-day and 14-day period preceding injury, with TL values fluctuating both above and below those of the control periods. These findings suggest that short-term TL alone may not be a reliable indicator of HSI risk and highlight the importance of a multifactorial approach to injury prevention. While TL monitoring remains a valuable tool, it should be interpreted in context with other individual and contextual factors. Future research with larger cohorts is needed to confirm these findings and explore more sensitive markers of injury risk.

## Supplementary material

10.1136/bmjsem-2025-002649 online supplemental material 1

## Data Availability

All data relevant to the study are included in the article or uploaded as online supplemental information.
