# Supplementary material for: Hamstring injury risk in male professional football: do external training loads play a role?
Source: BMJ Open Sport Exerc Med. 2025 Sep 9;11(3):e002649. doi: 10.1136/bmjsem-2025-002649 (PMC12421611; doi:10.1136/bmjsem-2025-002649)
Supplement: online supplemental material 1 [file bmjsem-11-3-s001.docx]

Table 1. Comparison of a seven day period leading up to injury and a control period of seven days by calculating non-overlap of all pairs (NAP). ID: Individual, TD: total distance (>0 km/h), VIA:very intense accelerations ( >3.00 m/s²), VID: very intense decelerations (<-3.00 m/s²), HSRd: high-speed running distance (>19,8 km/h), MSd: maximal sprint distance (> 29,8 km/h) and VMax: maximal velocity.

| ID | Distance | HSR | Zon 5 | Deacc | Acc | Max_Topp |
| --- | --- | --- | --- | --- | --- | --- |
| 1 | 0.36 | 0.40 | 0.50 | 0.48 | 0.45 | 0.40 |
| 2 | 0.43 | 0.41 | 0.47 | 0.41 | 0.43 | 0.49 |
| 3 | 0.45 | 0.55 | 0.55 | 0.53 | 0.49 | 0.61 |
| 4 | 0.36 | 0.47 | 0.57 | 0.34 | 0.40 | 0.46 |
| 5 | 0.49 | 0.45 | 0.49 | 0.50 | 0.52 | 0.46 |
| 6 | 0.50 | 0.50 | 0.50 | 0.47 | 0.47 | 0.49 |
| 7 | 0.54 | 0.57 | 0.50 | 0.65 | 0.67 | 0.60 |
| 8 | 0.55 | 0.53 | 0.43 | 0.42 | 0.46 | 0.51 |
| 9 | 0.51 | 0.49 | 0.50 | 0.52 | 0.58 | 0.49 |
| 10 | 0.46 | 0.44 | 0.40 | 0.51 | 0.49 | 0.45 |
| 11 | 0.37 | 0.39 | 0.36 | 0.35 | 0.40 | 0.28 |
| 12 | 0.51 | 0.51 | 0.49 | 0.53 | 0.59 | 0.49 |
| 13 | 0.29 | 0.29 | 0.43 | 0.27 | 0.27 | 0.18 |
| 14 | 0.33 | 0.43 | 0.43 | 0.37 | 0.32 | 0.37 |
| 15 | 0.27 | 0.27 | 0.57 | 0.24 | 0.28 | 0.22 |
| 16 | 0.57 | 0.55 | 0.51 | 0.50 | 0.50 | 0.57 |
| 17 | 0.57 | 0.57 | 0.51 | 0.58 | 0.62 | 0.57 |
| 18 | 0.51 | 0.53 | 0.53 | 0.51 | 0.54 | 0.56 |
| 19 | 0.39 | 0.35 | 0.42 | 0.42 | 0.37 | 0.39 |
| 20 | 0.45 | 0.42 | 0.46 | 0.48 | 0.53 | 0.45 |
| 21 | 0.47 | 0.43 | 0.43 | 0.37 | 0.39 | 0.39 |
| 22 | 0.65 | 0.65 | 0.50 | 0.50 | 0.50 | 0.63 |
| 23 | 0.37 | 0.29 | 0.43 | 0.50 | 0.50 | 0.29 |
| 24 | 0.59 | 0.53 | 0.43 | 0.60 | 0.55 | 0.49 |
| 25 | 0.45 | 0.51 | 0.41 | 0.67 | 0.64 | 0.51 |
|  |  |  |  |  |  |  |
| Group average  95 % CI | 0.47  (0.42, 0.53) | 0.46  (0.41, 0.52) | 0.48  (0.42, 0.53) | 0.48  (0.43, 0.54) | 0.49  (0.44, 0.55) | 0.46  (0.41, 0.52) |

Table 2. Comparison of a 14 day period leading up to injury and a control period of 14 days by calculating non-overlap of all pairs (NAP). ID: Individual, TD: total distance (>0 km/h), VIA:very intense accelerations ( >3.00 m/s²), VID: very intense decelerations (<-3.00 m/s²), HSRd: high-speed running distance (>19,8 km/h), MSd: maximal sprint distance (> 29,8 km/h) and VMax: maximal velocity.

| ID | Distance | HSR | Zon 5 | Deacc | Acc | Max_Topp |
| --- | --- | --- | --- | --- | --- | --- |
| 1 | 0.48 | 0.47 | 0.43 | 0.47 | 0.47 | 0.46 |
| 2 | 0.48 | 0.50 | 0.53 | 0.53 | 0.52 | 0.49 |
| 3 | 0.44 | 0.56 | 0.51 | 0.55 | 0.49 | 0.54 |
| 4 | 0.48 | 0.51 | 0.50 | 0.60 | 0.58 | 0.54 |
| 5 | 0.56 | 0.53 | 0.50 | 0.55 | 0.54 | 0.60 |
| 6 | 0.40 | 0.45 | 0.48 | 0.42 | 0.40 | 0.42 |
| 7 | 0.41 | 0.39 | 0.43 | 0.40 | 0.38 | 0.38 |
| 8 | 0.51 | 0.52 | 0.50 | 0.47 | 0.47 | 0.49 |
| 9 | 0.55 | 0.58 | 0.50 | 0.56 | 0.57 | 0.54 |
| 10 | 0.42 | 0.35 | 0.45 | 0.50 | 0.48 | 0.35 |
| 11 | 0.41 | 0.46 | 0.54 | 0.47 | 0.43 | 0.45 |
| 12 | 0.65 | 0.64 | 0.53 | 0.64 | 0.70 | 0.61 |
| 13 | 0.48 | 0.48 | 0.54 | 0.48 | 0.48 | 0.54 |
| 14 | 0.46 | 0.43 | 0.49 | 0.42 | 0.47 | 0.45 |
| 15 | 0.47 | 0.49 | 0.54 | 0.47 | 0.51 | 0.47 |
| 16 | 0.29 | 0.31 | 0.49 | 0.31 | 0.31 | 0.33 |
| 17 | 0.49 | 0.51 | 0.54 | 0.49 | 0.47 | 0.53 |
| 18 | 0.50 | 0.50 | 0.48 | 0.50 | 0.50 | 0.52 |
| 19 | 0.60 | 0.63 | 0.57 | 0.62 | 0.66 | 0.66 |
| 20 | 0.54 | 0.51 | 0.56 | 0.49 | 0.50 | 0.55 |
| 21 | 0.44 | 0.40 | 0.50 | 0.51 | 0.45 | 0.46 |
| 22 | 0.61 | 0.58 | 0.50 | 0.50 | 0.50 | 0.59 |
| 23 | 0.53 | 0.49 | 0.54 | 0.50 | 0.50 | 0.56 |
| 24 | 0.52 | 0.51 | 0.44 | 0.46 | 0.49 | 0.49 |
| 25 | 0.43 | 0.51 | 0.51 | 0.44 | 0.45 | 0.50 |
|  |  |  |  |  |  |  |
| Group average | 0.49 | 0.49 | 0.50 | 0.49 | 0.49 | 0.50 |
| 95 % CI | (0.45, 0.53) | (0.45, 0.53) | (0.46, 0.54) | (0.45, 0.53) | (0.45, 0.53) | (0.46, 0.54) |
